# Leclercia adecarboxylata cellulitis in a neutropenic Thai child with acute myeloid leukemia

**DOI:** 10.1016/j.idcr.2026.e02688

**Published:** 2026-07-16

**Authors:** Theppharit Thiamprasert, Paranyu Ruangsirinusorn, Yujinda Lektrakul, Chareeya Thanee

**Affiliations:** a Division of Infectious Diseases, Department of Pediatrics, Sunpasitthiprasong Hospital, Ubon Ratchathani, Thailand; b Division of Hematology-oncology, Department of Pediatrics, Sunpasitthiprasong Hospital, Ubon Ratchathani, Thailand

**Keywords:** Leclercia adecarboxylata, Cellulitis, Acute myeloid leukemia, Neutropenia, Pediatric infection

## Abstract

**Introduction:**

*Leclercia adecarboxylata* is a rare opportunistic Gram-negative organism that has increasingly been recognized as a pathogen in immunocompromised patients. In pediatric populations, infections are uncommon and rarely reported, particularly in Thailand.

Case presentation: We report a 3-year-old Thai boy with acute myeloid leukemia in the induction phase who presented with febrile neutropenia and right knee cellulitis following minor trauma. Pus culture grew *L. adecarboxylata*, which was confirmed by MALDI-TOF mass spectrometry, while blood culture remained negative. Despite profound neutropenia, the infection remained localized without bacteremia. The patient was treated with intravenous ceftazidime and amikacin, followed by oral amoxicillin-clavulanic acid, for a total of 14 days, with complete clinical recovery.

**Conclusion:**

This case highlights *L. adecarboxylata* as a potential pathogen in immunocompromised children with skin and soft tissue infections, particularly following environmental exposure. Clinicians should be aware that *L. adecarboxylata* may cause clinically significant skin and soft tissue infections and, in high-risk patients, has also been associated with invasive infections, including bacteremia.

## Highlights

• *Leclercia adecarboxylata* causes cellulitis in a neutropenic child.
• Trauma with soil exposure precedes infection.
• Infection remains localized without bacteremia.
• MALDI-TOF enables accurate organism identification.
• Targeted antimicrobial therapy leads to full recovery.

## Introduction

*Leclercia adecarboxylata* (*L. adecarboxylata*), previously known as *Escherichia adecarboxylata*, is a motile, aerobic, gram-negative bacillus belonging to the *Enterobacteriaceae* family. It was first reported in 1962 by H. Leclerc [1]. It is commonly isolated from environmental sources such as water and soil. Although historically regarded as a low-virulence organism, it has been increasingly recognized as a human pathogen, causing mixed infections in immunocompetent patients and single-pathogen infections in immunocompromised hosts [2].

In immunocompetent children, *L. adecarboxylata* can cause various infections, including folliculitis, cellulitis, wound infection, abscess, and septic arthritis, usually following penetrating injury or laceration wounds and typically resulting in favorable outcomes [3], [4], [5], [6]. In contrast, in immunocompromised children *L. adecarboxylata* has been reported to cause a variety of infections, including cellulitis, peritonitis, and bacteremia [7], [8], [9], [10], [11]. However, reports in immunocompromised children remain limited and are associated with a higher risk of invasive disease. In Thailand, infections caused by this organism are rare; to our knowledge, only one pediatric case has been reported, describing cellulitis in an immunocompetent child following a penetrating injury [12].

Although most isolates remain susceptible to commonly used antibiotics, emerging resistant strains have been described [2]. Given its potential to cause clinically significant infection and the scarcity of pediatric reports, we describe this case to raise awareness of *L. adecarboxylata* as an uncommon but important pathogen in vulnerable pediatric populations.

## Case presentation

**Fig. 1:**
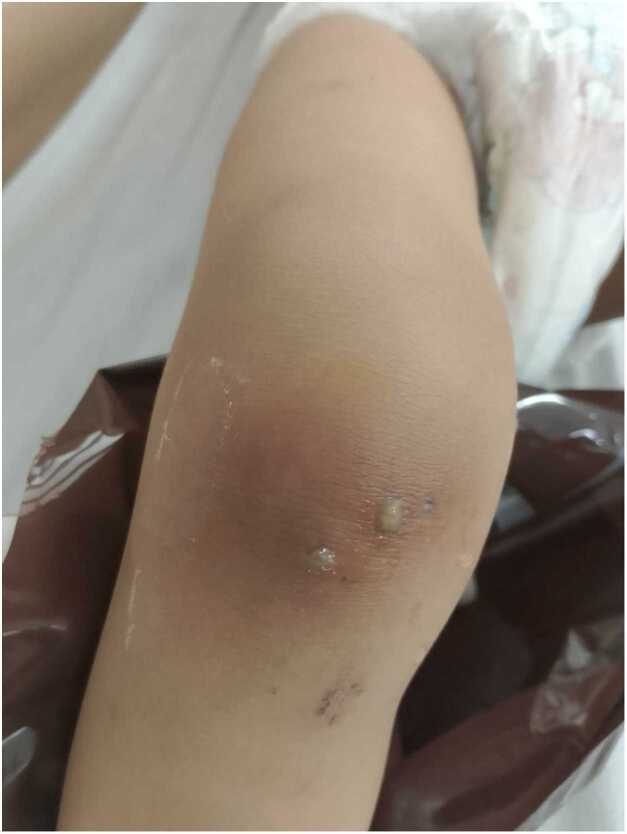
Patient’s lesion on arrival.

A 3-year-old Thai boy with underlying acute myeloid leukemia on chemotherapy in his induction phase with resolving invasive pulmonary aspergillosis was admitted with fever and swelling of the right knee. He had completed chemotherapy with etoposide and cytarabine 2 days prior to this admission. One day after discharge, he sustained a minor fall on his right knee at his house onto dry gravel and soil ground. That night, his mother noticed erythema, swelling, and tenderness at the site, accompanied by fever. His mother therefore brought him to seek medical care. On arrival, he appeared ill and febrile with a temperature of 40.8 °C, heart rate 150 /min, respiratory rate 26 /min, and blood pressure 113/54 mmHg. Examination revealed erythema, tenderness, and swelling of the right knee with seropurulent discharge (Fig. 1). There was no involvement of the joint or surrounding structures.

Initial laboratory results showed hemoglobin 6.7 g/dL, hematocrit 19.8%, total white blood cell count 280 cells/µL with 6% neutrophils (absolute neutrophil count 17 cells/µL), lymphocytes 93%, and platelets 3000 cells/µL. X-ray of his knees was normal on both sides, no signs of acute osteomyelitis or effusion. He was diagnosed with febrile neutropenia and right knee cellulitis and was started empirically on intravenous piperacillin–tazobactam and cloxacillin. Only blood culture was obtained on that day, and no pus culture was collected.

**Fig. 2:**
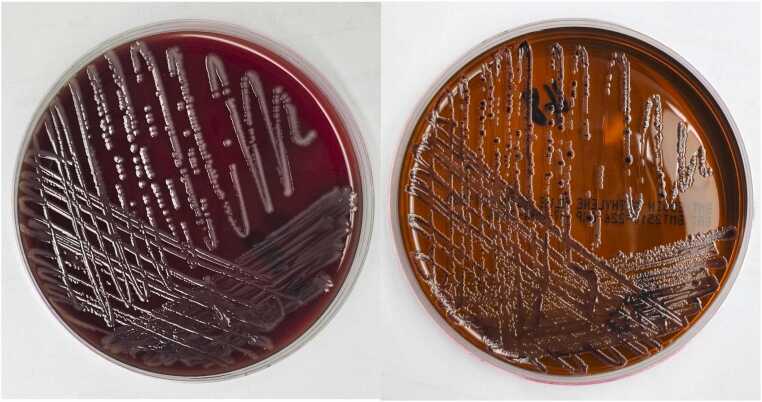
Colonies of *Leclercia adecarboxylata* on blood agar and BMT eosin methylene blue agar.

**Fig. 3:**
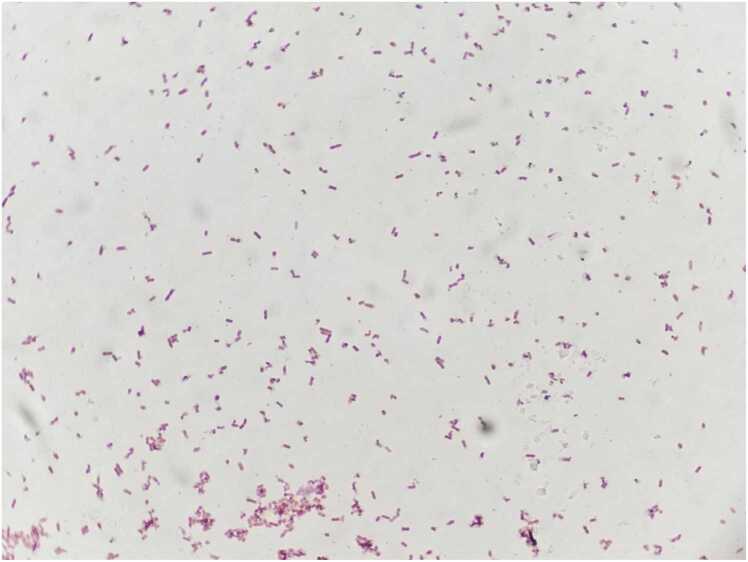
Gram stain from the culture plate showing gram-negative bacilli.

Despite treatment, he remained febrile and continued to have purulent drainage by hospital day 3. Pus was collected for culture; no organisms were observed on Gram stain. After 5 days of hospitalization, his fever persisted around 39 °C. Pus culture subsequently yielded *Leclercia adecarboxylata*, identified by biochemical testing and confirmed by MALDI-TOF mass spectrometry (Fig. 2, Fig. 3) and blood culture showed no growth. Antimicrobial susceptibility testing was performed using the VITEK 2 system, as shown in Table 1.

**Table 1:** Susceptibility testing of *Leclercia adecarboxylata*.

**Antibiotics**	**MIC**	**Interpretation**
Amoxicillin/Clavulanate	≤ 2	susceptible
Cefoperazone/Sulbactam	≤ 8	susceptible
Piperacillin/Tazobactam	≤ 4	susceptible
Ceftazidime	≤ 0.12	susceptible
Ceftriaxone	≤ 0.25	susceptible
Amikacin	≤ 1	susceptible
Gentamicin	≤ 1	susceptible
Ciprofloxacin	≤ 0.5	intermediate
Trimethoprim/Sulfamethoxazole	≤ 20	susceptible

**Fig. 4:**
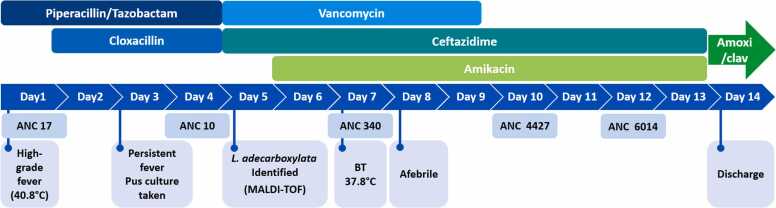
Timeline of hospitalization and treatment. ANC; absolute neutrophil count, BT; body temperature.

The antibiotic regimen was escalated to intravenous ceftazidime (150 mg/kg/day) and vancomycin (50 mg/kg/day) on day 5 of hospitalization due to persistent high-grade fever, swelling and erythema of the right knee. Amikacin (15 mg/kg/day) was added on day 6. On day 9 of hospitalization, as his clinical condition improved, vancomycin was discontinued following discussion with the pediatric infectious diseases team. The patient’s fever resolved without the need for incision and drainage, and the cellulitis gradually improved. A two-week course of antimicrobial therapy (ceftazidime and amikacin for 9 days, followed by oral amoxicillin-clavulanic acid 45 mg/kg/day for 5 days) was completed with full recovery and no complications (Fig. 4).

## Discussion

*Leclercia adecarboxylata* is a ubiquitous gram-negative bacillus that has traditionally been considered a low-virulence organism but is increasingly recognized as an opportunistic pathogen, particularly in immunocompromised hosts [2]. Representative published pediatric cases are summarized in Table 2. Reported clinical manifestations include folliculitis, abscess, septic arthritis, cellulitis, spontaneous bacterial peritonitis, and bacteremia. Among immunocompetent children, most reported infections followed penetrating trauma or environmental exposure and remained localized skin and soft tissue infections. ^3–6,12^ In contrast, immunocompromised children are more likely to develop invasive or monomicrobial infections [7], [8], [9] and may result in severe outcomes [10], [11].

**Table 2:** Summary of published pediatric *Leclercia adecarboxylata* infections.

**Reference**	**Age**	**Underlying condition**	**Immunocompromised** **Status**	**Syndrome**	**Trauma**	**Outcome**
Broderick et al. 2019 [3]	12 years	Healthy	No	Folliculitis	No	Recovered
Hurley et al. 2015 [4]	2 years	Healthy	No	Infected wound, Cellulitis	Yes	Recovered
Grantham et al. 2015 [5]	9 years	Healthy	No	Infected wound, Cellulitis	Yes	Recovered
Arasu et al. 2022 [6]	7 years	Healthy	No	Septic arthritis	Yes	Recovered
	3 years	Healthy	No	Septic arthritis	Yes	Recovered
Shah et al. 2011 [7]	8 years	Acute Lymphoblastic Leukemia	Yes	Ecthyma gangrenosum, Cellulitis	No	Recovered
Hassan et al. 2020 [8]	7 years	Nephrotic syndrome	Yes	Peritonitis	No	Recovered
Myers et al. 2012 [9]	16 days	Premature infant 26 weeks	Yes	Bacteremia	No	Recovered
Nelson et al. 2013 [10]	31 days	Premature infant 24 weeks	Yes	Bacteremia	No	Died
Bronte et al. 2022 [11]	4 days	Premature infant 24 weeks	Yes	Bacteremia	No	Died
Vasaruchapong et al. 2024 [12]	12 years	Healthy	No	Infected wound, Cellulitis	Yes	Recovered
Present case	3 years	Acute Myeloid Leukemia	Yes	Cellulitis	Yes	Recovered

Compared with the pediatric literature, adult cases are considerably more numerous and encompass a broader spectrum of clinical presentations, including wound infections, cellulitis, pharyngeal and peritonsillar abscesses, endocarditis, urinary tract infections, pneumonia, and bacteremia [2], [13]. Nevertheless, the overall pattern appears similar, with localized skin and soft tissue infections predominating in immunocompetent individuals and invasive or monomicrobial infections occurring more frequently in immunocompromised hosts. In addition, several adult reports have highlighted central venous catheters as an important risk factor for *L. adecarboxylata* bloodstream infection, regardless of immune status [13].

In our case, underlying acute myeloid leukemia and the profound neutropenia during the induction phase were critical predisposing factors. The history of trauma-associated environmental exposure likely resulted in inoculation of environmental microorganisms, thereby broadening the differential diagnosis to include unusual Gram-negative pathogens, among which *L. adecarboxylata* is one reported possibility. This is consistent with previous reports describing trauma-associated environmental exposure as a risk factor for infection [4], [5], [6].

Despite severe neutropenia, the infection in our case remained localized in the absence of bacteremia, illustrating the variable clinical spectrum of *L. adecarboxylata* infection even among immunocompromised children.

Accurate microbiological identification and antimicrobial susceptibility testing are essential in infections caused by uncommon organisms such as *L. adecarboxylata*. In our case, the organism was identified using MALDI-TOF mass spectrometry. Although routine culture media, including blood agar and MacConkey agar, are sufficient for isolation of *L. adecarboxylata*, accurate species-level identification may be challenging because conventional biochemical methods can misidentify the organism as other members of the Enterobacterales owing to overlapping biochemical characteristics [13]. MALDI-TOF mass spectrometry enabled rapid, accurate species-level identification, thereby facilitating recognition of this uncommon pathogen in clinical practice [2], [13].

Antimicrobial susceptibility testing demonstrated broad susceptibility to commonly used antibiotics, consistent with previous reviews, which reported that *L. adecarboxylata* remains susceptible to most β-lactams, aminoglycosides, fluoroquinolones, and trimethoprim-sulfamethoxazole, although resistant isolates carrying ESBL or carbapenemase genes have occasionally been reported [2], [13].

The patient was initially treated empirically with piperacillin–tazobactam and cloxacillin for febrile neutropenia, with coverage targeting *Pseudomonas aeruginosa* and methicillin-susceptible *Staphylococcus aureus*. However, persistent fever with profound neutropenia and ongoing local inflammation prompted escalation to ceftazidime and amikacin to broaden Gram-negative coverage, including pathogens such as *Burkholderia pseudomallei* and ESBL-producing organisms, which are relevant in our specific regional setting. However, there is no evidence that combination antimicrobial therapy is routinely required for *L. adecarboxylata* infection itself. Because the isolate was susceptible to piperacillin–tazobactam, persistent fever early in the clinical course may have reflected profound neutropenia rather than microbiological treatment failure. Therefore, the subsequent clinical improvement cannot be attributed solely to modification of antimicrobial therapy. Instead, it was likely multifactorial, reflecting continued active antimicrobial treatment, broader Gram-negative coverage in the local epidemiologic context, and gradual recovery of the neutrophil count.

The optimal duration of antimicrobial therapy for cellulitis is typically around 5 days in uncomplicated cases [14]. However, in our patient, although no organism-specific treatment duration has been established for *L. adecarboxylata,* a total of 14 days of antimicrobial therapy was selected because of profound neutropenia, persistent fever, delayed clinical improvement, and the immunocompromised state, and was consistent with previously reported pediatric and adult cases [7], [12], [15].

This case highlights that *L. adecarboxylata* should be included in the differential diagnosis of soft tissue infections in neutropenic children, especially when there is a history of environmental exposure. Early recognition through advanced identification methods and appropriate antimicrobial therapy guided by susceptibility testing and clinical response are vital for ensuring favorable outcomes.

## Conclusion

*L. adecarboxylata* is an uncommon but clinically relevant pathogen in immunocompromised pediatric patients. Increased awareness of this organism in neutropenic children with trauma-associated cellulitis may facilitate early diagnosis and appropriate management, leading to favorable clinical outcomes.

## Authors’ contributions

T. T. contributed to data acquisition, data interpretation, and drafting and revision of the manuscript. P.R. and Y. L. contributed to data acquisition. C.T. contributed to revision of the manuscript. All authors have read and approved the final version of the manuscript to be submitted for journal publication.

## CRediT authorship contribution statement

**Chareeya Thanee:** Supervision. **Yujinda Lektrakul:** Resources, Data curation. **Paranyu Ruangsirinusorn:** Data curation. **Theppharit Thiamprasert:** Writing – original draft, Data curation.

## Ethics approval and consent to participate

The patient and his guardian provided informed consent for this publication. This case report was approved by the ethical committee of Sunpasitthiprasong Hospital, Ubon Ratchathani, Thailand. (0133/69 R on March 2, 2026).

## Ethics declaration

Written informed consent to take part in the study and to publish the article has been obtained from all participants or their legal representatives. The privacy rights of participants have been observed.

This study was performed in compliance with relevant laws, regulatory frameworks and guidelines where the research took place. This study was approved by the the ethical committee of Sunpasitthiprasong Hospital, Ubon Ratchathani, Thailand. (Approval No. 0133/69R).

This research follows the CARE guidelines and the CARE checklist.

## Funding

Open access funding provided by Research Development Fund, Sunpasitthiprasong Hospital. The authors received no funding for this work.

## Declaration of Generative AI and AI-assisted technologies in the writing process

During the preparation of this work the authors used ChatGPT to assist with language editing, including translation from Thai into formal academic English and grammatical correction. After using this tool/service, the authors reviewed and edited the content as needed and take full responsibility for the content of the published article.

## Declaration of Competing Interest

The authors declare that they have no known competing financial interests or personal relationships that could have appeared to influence the work reported in this paper.

## Data Availability

All data during this study are included in this article.
